# LamNI – an instrument for X-ray scanning microscopy in lamino­graphy geometry

**DOI:** 10.1107/S1600577520003586

**Published:** 2020-04-06

**Authors:** Mirko Holler, Michal Odstrčil, Manuel Guizar-Sicairos, Maxime Lebugle, Ulrich Frommherz, Thierry Lachat, Oliver Bunk, Joerg Raabe, Gabriel Aeppli

**Affiliations:** a Photon Science, Paul Scherrer Institut, Forschungsstrasse 111, 5232 Villigen PSI, Switzerland; b Large Research Facilities, Paul Scherrer Institut, Forschungsstrasse 111, 5232 Villigen PSI, Switzerland; c EnDes Engineering Partner AG, 4703 Kestenholz, Switzerland

**Keywords:** nano 3D imaging, ptychography, lamino­graphy, scanning microscopy

## Abstract

Across all branches of science, medicine and engineering, high-resolution microscopy is required to understand functionality. Although optical methods have been developed to ‘defeat’ the diffraction limit and produce 3D images, and electrons have proven ever more useful in creating pictures of small objects or thin sections, so far there is no substitute for X-ray microscopy in providing multiscale 3D images of objects with a single instrument and minimal labeling and preparation. A powerful technique proven to continuously access length scales from 10 nm to 10 µm is ptychographic X-ray computed tomography, which, on account of the orthogonality of the tomographic rotation axis to the illuminating beam, still has the limitation of necessitating pillar-shaped samples of small (*ca* 10 µm) diameter. Large-area planar samples are common in science and engineering, and it is therefore highly desirable to create an X-ray microscope that can examine such samples without the extraction of pillars. Computed lamino­graphy, where the axis of rotation is not perpendicular to the illumination direction, solves this problem. This entailed the development of a new instrument, LamNI, dedicated to high-resolution 3D scanning X-ray microscopy via hard X-ray ptychographic lamino­graphy. Scanning precision is achieved by a dedicated interferometry scheme and the instrument covers a scan range of 12 mm × 12 mm with a position stability of 2 nm and positioning errors below 5 nm. A new feature of LamNI is a pair of counter-rotating stages carrying the sample and interferometric mirrors, respectively.

## Abstract 2

A technical description of an instrument that allows 3D nano-imaging via ptychographic X-ray lamino­graphy is presented.

## 1. Introduction   

Computed tomography (CT) is a powerful technique for nondestructive 3D imaging (Grangeat, 2010 ▸). Common CT uses 2D projections acquired at different sample orientations, for which the sample is rotated around an axis perpendicular to the illumination direction. If a sufficient number of projections is measured over the full 180° range to fulfill the Crowther criterion (Crowther *et al.*, 1970 ▸), the recorded data can be regarded as complete and the resulting 3D image can reach isotropic resolution. Over the past decade, the resolution of X-ray tomography has drastically increased by employing lens-less imaging schemes, and many instruments dedicated to high-resolution X-ray tomography have been developed and built and are in routine use today (Silva *et al.*, 2017 ▸; Holler *et al.*, 2012 ▸, 2018 ▸; Nazaretski *et al.*, 2017 ▸; Schroer *et al.*, 2017 ▸; Celestre *et al.*, 2017 ▸; Klug *et al.*, 2018 ▸).

Although high-resolution tomographic imaging is clearly important for many scientific projects, it requires the sample to be accessible from the full angular range. Thus, cylindrically shaped specimens are ideal for tomography, which, in the case of X-ray nanotomographic imaging, often requires small samples and so typically tedious, complex and destructive preparation.

If an object is rather flat and large in two dimensions, it can still be measured using tomography. Assuming the object has a thickness *t*, the effective thickness (*t*_eff_) that the radiation has to penetrate depends on the projection angle α and increases according to *t*_eff_ = *t*/cosα, meaning that, at larger angles, the effective thickness will rapidly increase. This leads to problems due to the high absorption and limitations in depth of field, not to mention the obscurations and collisions risked when a large extended object is rotated in this configuration. For these flat objects only a subset of orientations are available, resulting in a missing wedge of information in reciprocal space which leads to reconstruction artefacts and non-isotropic resolution in the 3D reconstruction (Bartesaghi *et al.*, 2008 ▸).

**Figure 1:**
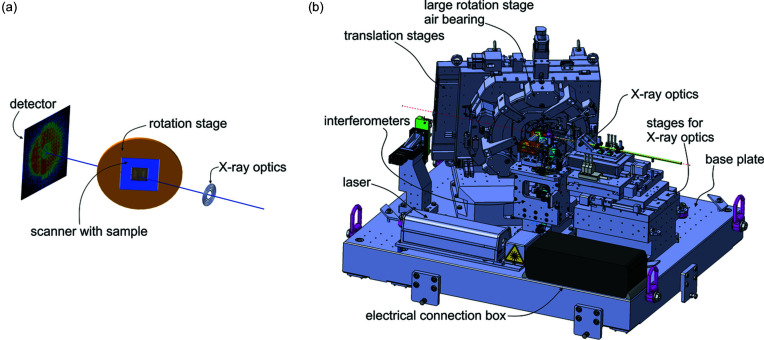
(*a*) Schematic of the lamino­graphy geometry. (*b*) Overview of the LamNI instrument. All components are installed on a common base plate with the dimensions 110 cm × 130 cm.

For such samples the situation can be improved by using lamino­graphy (Hasenkamp, 1974 ▸; Helfen *et al.*, 2005 ▸, 2009 ▸, 2011 ▸) instead of tomography. Here the axis of rotation is inclined with respect to the incoming radiation. Although this also does not allow reconstruction of a 3D dataset with isotropic resolution, the missing information is reduced from a wedge to a cone, meaning more information can be recovered. Corresponding projections with an angular offset of 180° are not just mirrored anymore, and a measurement requires projections collected over the full 360° angular range in order to cover the reciprocal space; see also Fig. 1 in our recent work (Holler *et al.*, 2019 ▸). For planar samples, laminography projections have the advantage of having an effective thickness that remains constant throughout the measurement, which enables equal measurement conditions and comparable quality for all projection angles.

In our case, we use X-ray ptychography for imaging, which, at several keV photon energies, allows, depending on the material composition, penetration of samples with a thickness of up to about 100 µm, while also allowing for sub-10 nm 2D resolution (Vila-Comamala *et al.*, 2011 ▸; Thibault *et al.*, 2008 ▸), although currently only in smaller volumes. Ptychography is a scanning coherent diffraction imaging technique where a sample is illuminated by a spatially confined coherent beam and a far-field diffraction pattern is recorded for each scan position. Partially overlapping regions in the adjacent scanning positions allow reliable solution of the phase problem using iterative reconstruction algorithms, thereby reconstructing both the 2D complex-valued object transmissivity and the illumination probe. In theory, ptychography can provide diffraction-limited resolution, assuming that the illumination probe is constant and sample positions are known with higher accuracy than the target resolution. In past work we developed and built two instruments for ptychographic X-ray computed nanotomography (PXCT) (Holler *et al.*, 2012 ▸, 2014 ▸, 2018 ▸) for which dedicated laser interferometry (Holler & Raabe, 2015 ▸) was implemented as position metrology compatible with the required sample translation and rotation. In these instruments, the sample is mounted on a pin (Holler *et al.*, 2017*a* ▸) and inserted into a sample holder which also serves as a reference mirror used in position metrology. This allowed an isotropic 3D resolution of 14.6 nm for a cylindrically shaped sample of 10 µm diameter extracted from an integrated circuit (Holler *et al.*, 2017*b* ▸).

We present here a new instrument, LamNI (laminographic nano-imaging), that uses ptychographic X-ray lamino­graphy (PyXL) for imaging, meaning it combines ptychography for the acquisition of high-resolution projections and lamino­graphy for gaining access to the third spatial dimension. The full 2D scan area that can be covered via stitching is 12 mm × 12 mm. The previously mentioned laser interferometry (Holler & Raabe, 2015 ▸) for measuring the sample position is not compatible with such large scan ranges and thus a different concept was developed for LamNI. A first demonstration of the 3D imaging capabilities for an integrated circuit sample can be found in our recent work (Holler *et al.*, 2019 ▸). In the latter, a CMOS circuit manufactured in the 16 nm node was measured at 13 nm voxel size covering a volume of 3800 × 3800 × 600 elements. The 3D resolution was estimated to be 18.9 nm. PyXL has also proven useful for measurements with magnetic contrast. Donnelly *et al.* (2020 ▸) have demonstrated time-resolved measurements of magnetization dynamics in a microdisc using LamNI, where the lamino­graphy geometry has also proven to be advantageous compared with tomography in the ease of implementing magnetic field excitations. The spatial and temporal resolution achieved was 50 nm and 70 ps, respectively, the latter limited by the timing structure of the synchrotron source.

## 2. Basic arrangement of the components of LamNI   

The basic arrangement of the individual sub-systems is similar to the tomography instruments previously described (Holler *et al.*, 2012 ▸, 2014 ▸, 2018 ▸). The X-ray beam enters the setup depicted in Fig. 1 ▸ from the right and first passes through the beam-defining optical elements. We use a Fresnel zone plate (FZP) to define the beam on the sample (Gorelick *et al.*, 2011*a* ▸,*b* ▸; Odstrčil *et al.*, 2019*a* ▸). The optics unit therefore consists of three elements: the FZP, a central stop (CS) before the FZP and an order-sorting aperture (OSA) after the FZP, which are used to block the zero and higher diffraction orders of the FZP. The focused beam then interacts with the sample and the transmitted light propagates though a flight tube, either filled with helium or under vacuum, to the detector.

In our right-handed coordinate system the X-ray beam propagates along the *z* direction and the vertical direction is denoted by *y*. The sample stage is tilted the following way: starting with the axis of rotation for lamino­graphy parallel to the X-ray propagation direction, the stage is first tilted around the *x* axis by 15° and then tilted around the *y* axis by 60°. The resulting lamino­graphy angle (*i.e.* the angle between the X-ray beam and the rotation axis) is 61°, in contrast to the angle of 90° that corresponds to the case of conventional tomography. Since mechanical systems also require a certain thickness to be stable, the open aperture of a stage mechanism grows rapidly with the angle for the radiation to pass. This means that the mechanical guiding elements of the translation and rotation mechanism of the sample are spaced far apart, which naturally reduces angular error motions and keeps the lamino­graphy angle constant throughout the measurement.

**Figure 2:**
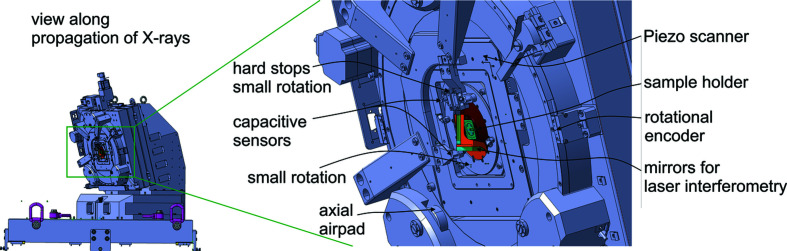
View of the lamino­graphy stage along the X-ray beam propagation direction.

The sample position is measured interferometrically with respect to the beam-defining FZP in the *x* and *y *directions. Flat mirrors positioned on the sample stage and FZP holder are used for interferometry in combination with double-pass interferometers [see Fig. 2 ▸(*b*) of Holler & Raabe (2015 ▸)]. The interferometers use a beam of 6 mm diameter and the laser source and readout electronics are from Zygo Inc. The tilt of the sample stage around the *x* axis of 15° created the required space to implement the vertical interferometry below the sample stage. Geometrically, the interferometer measurement points and the X-ray beam intersect the sample plane at the measurement position. Therefore, the geometrical interferometer errors caused by angular error motion are minimized.

## 3. The sample stage   

The sample stage consists of several stacked stages. Starting from the sample, these stages include a piezoelectric scanner offering two translational degrees of freedom and a travel range of 100 µm in each direction (Npoint NPXY100-216). This stage is used for fast and accurate sample positioning for the ptychography scans. It is equipped with internal capacitive sensors, but the scans are performed in a closed loop relative to the exteroceptive interferometric position measurement. The piezoelectric scanner is mounted on a large rotation stage covering an angular range of 365° and equipped with a dual encoding system from Renishaw (Tonic). The effective radius of the bearing system of the rotation stage is 175 mm, achieving motion with low angular error. The bearing system used is based on air-bearing pads and hence offers very reproducible motion. However, the stiffness of the air-bearings is typically lower than that of mechanical ball-bearing systems. To improve mechanical stiffness and reproducibility even further, the air pressure is removed after rotating and the rotation stage is effectively transformed into a metal plate mechanically clamped by the pre-tensions of the air-pads.

The sample holder is directly installed on the piezoelectric scanner and samples are mounted from the rear side of the stage. The imaging range of 12 mm × 12 mm is too large for using tracking interferometry and spherical mirrors (Holler & Raabe, 2015 ▸), so a solution using flat mirrors is required. This poses a problem: such reference mirrors have to be installed on the piezoelectric scanner so that they move with the sample during the scan, but, while the Piezo stage rotates during lamino­graphy data acquisition, the interferometry mirrors must stay perpendicular to the interferometer beam in order to maintain the position signal. The solution employed is a second rotation stage mounted on the piezoelectric scanner, which carries the mirrors used for interferometry. This rotation stage is based on a ball-bearing system and it prevents the mirrors from turning. It is passively moved by a lever arm as explained in more detail later. Small rotation stages typically suffer from larger angular error motion compared with systems with a large diameter. However, in the present configuration only the alignment of the mirrors is affected by such error motion which could introduce a scaling factor between the sample position and the interferometer reading. Such a scaling factor can be calibrated because error motions are mostly repeatable. Meanwhile, the lamino­graphy projection angle through the sample remains accurate and constant because the sample is mounted on the large rotation stage. The arrangement can also be seen in Fig. 2 ▸, showing a view of the sample stage along the X-ray propagation direction. Capacitive sensors are used to measure all angular motions of the mirror anti-rotation stage (Figs. 2 ▸ and 3 ▸). These data can be used to compute the geometrical changes of the alignment of the mirrors and simultaneously correct the position data used in the ptychographic reconstruction. After a rotation, the ball-bearing system of the mirror rotation stage can lead to a translational error in the 1 µm range between the mirrors and the sample when changing to a new rotation angle. This is not critical because, as in the previously described tomography instruments (Holler *et al.*, 2012 ▸, 2014 ▸, 2018 ▸), the projections are aligned and computationally combined into a 3D data set without relying on the interferometry data (Odstrčil *et al.*, 2019*b* ▸). The instrument mainly ensures distortion-free and accurate projections in the absence of a rotation.

Without the large rotation stage, but instead with the sample mounted directly on a small rotation stage on a fixed angle piezo scanner, the setup would appear simpler. However, motion-induced angular errors would then affect the lamino­graphy projection geometry, which complicates the alignment and combination of the projections into a 3D dataset. This becomes more critical as the ratio of sample thickness to resolution increases. Mounting a large rotation stage on the scanner with better rotational behavior instead is also not desirable because for a fast motion the mobile mass on the scanner should be minimized.

**Figure 3:**
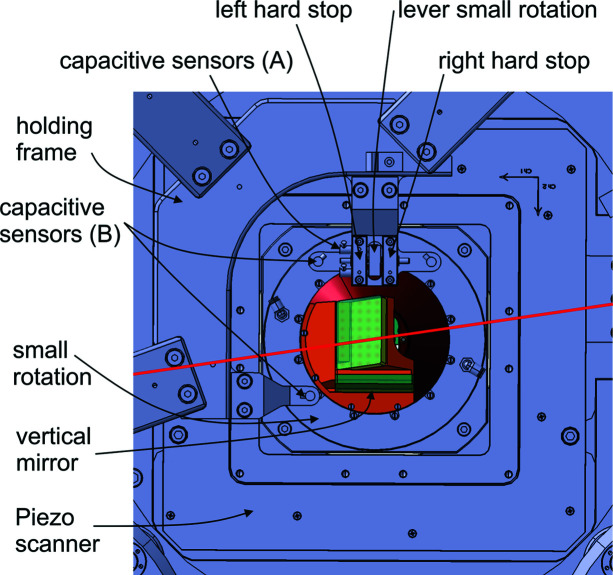
View of the sample scanner perpendicular to the sample plane.

An ‘anti-rotation’ stage for the mirrors on the scanner must always move synchronously in the opposite direction of the large rotation stage. If the motion is not fully synchronized, the mirrors will be misaligned too much and the interferometry signal will be lost. The small rotation stage does not have an active drive. Instead the drive mechanism used is a simple lever attached to the small rotation stage mounted between two fixed hard stops installed at the base of the large rotation stage (see Fig. 3 ▸). When rotating in any direction with the large rotation stage, the lever touches a hard stop on either side and keeps the small rotation stage aligned (see also Video S1 of the supporting information).

An additional requirement is to immobilize the small rotation stage during a scan. For this purpose, it is equipped with a pneumatic brake mechanism. During a ptychography scan, the stage is clamped and fixed in position. The complication is that there should always be enough free travel between the hard stops and the lever for a scan to be conducted, because the small rotation stage, and therefore the lever, are moved by the piezo-scanner. Touching a hard stop during the scan while having the small rotation stage clamped could damage the piezoelectric scanner and cause error motions.

There are two capacitive sensors, shown in Fig. 3 ▸ as capacitive sensor (A), with a measurement range of 250 µm (Lion precision) implemented in one hard stop. One sensor would be sufficient to measure the distance between the lever and the hard stop. However, the configuration with two sensors allows measurement of the angle of the small rotation stage. This angle can be slightly different compared with the angle of the large rotation since the stages will never be perfectly centered to each other. When rotating to a new projection angle, the procedure is as follows: the piezoelectric scanner is centered within its own reference frame, *i.e.* to the internal capacitive sensors. At that point, feedback to the interferometry is switched off. The brake mechanism of the small rotation is released and the air pressure of the large rotation stage is activated. The large stage is rotated to the newly desired angle using the encoder system as position feedback. When the position is reached, the small rotation stage is clamped. The control of the large rotation stage switches the position feedback to the angular measurement of the two capacitive sensors (A) and then aligns the small rotation stage to always the same angular position at which the lever of the small rotation stage is approximately centered between the hard stops. Two things are then achieved: the small rotation stage and thereby the mirrors are aligned to a reproducible angle and only the angular error motions of the small rotation along the two other angular directions have to be considered for correcting the interferometry position data for ptychographic reconstructions [Fig. 3 ▸, capacitive sensors (B)]. In addition, having the lever in the center of the hard stops gives freedom to the piezoelectric stage to move between the hard stops without colliding. As a safety mechanism, the position of the capacitive sensors is continuously supervised to ensure that the distance of the lever to the hard stop is never too small. To prevent a collision the small rotation stage is automatically un-clamped. In the last step, the axial air bearing of the large rotation is also switched off to increase the mechanical stiffness of the stage. After the centering procedure the large rotation stage is of course at a slightly different angle than initially intended. The latest readout of the angular encoder is stored and used for the lamino­graphic reconstruction. Video S1 shows the rotational mechanism in operation, rotating from 0° to 90°.

The large rotation stage is mounted on two linear stages that allow coarse translation for following a region of interest while rotating, and also for stitching several areas that have been scanned. A stitching example is shown in Fig. S1 of the supporting information. The linear stages and the rotation stages are driven by stepper motors and were developed and built by Steinmeyer Mechatronik, Dresden, Germany. While for the rotation stage a rotational encoder is used, during an X-ray measurement the translations of the large stages are performed with feedback to the interferometer position data with 0.1 µm resolution. During such motion, the piezo-scanner is centered via its internal capacitive sensors. Using the interferometer as an encoder for the large stages reduces the mechanical drift of the setup to only the intereorometric dead-path, *i.e.* the mirror-to-sample distance.

## 4. The X-ray optics unit   

**Figure 4:**
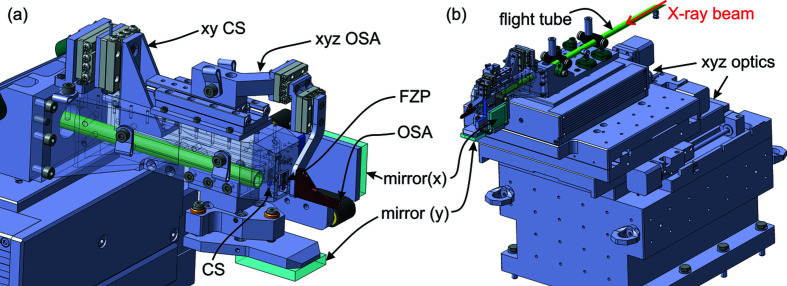
Optics unit. (*a*) Close-up of the optical holders. (*b*) Overview including the *xyz* optics stages and base block.

The X-ray optics unit is constructed using three linear stages (Steinmeyer Mechatronik) that are stepper motor driven and carry the entire optical unit, see Fig. 4 ▸(*b*). The FZP is directly mounted at the front of the optics holder [see Fig. 4 ▸(*a*)]. These optics *xyz* stages are the only means to move and align the FZP. The CS and OSA need to be aligned with respect to the FZP. The CS is mounted via two linear stages (*xy*; Smaract GmbH) and the OSA is mounted on three linear stages (*xyz*; Smaract GmbH). Both of these mechanisms are installed on the optics holder, so they move together with the optics stages. Two flat mirrors are attached via manual mirror aligners for the interferometric measurement of the optics position. A flight tube filled with helium in front of the optical elements allows a simple setup at the beamline. It extends out of the LamNI enclosure, and in the setup it ends in front of the CS.

## 5. Operation at the beamline   

The setup is used at the cSAXS beamline of the Swiss Light Source at the Paul Scherrer Institut. The entire setup was brought to the beamline fully assembled on its baseplate. Its total weight is 1 metric ton. It can be lifted by the two cranes in the experimental hutch of the beamline and was placed onto the experimental table resting on damping plates (Isoloc Schwingungstechnik GmbH, IPL10).

**Figure 5:**
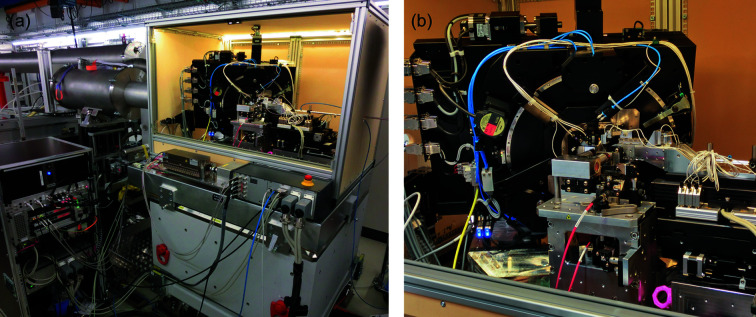
LamNI installed at the cSAXS beamline with the doors of the enclosure removed. (*a*) Overview with the flight tube and control rack. (*b*) Enlarged image.

Fig. 5 ▸ shows the setup installed at the beamline. The X-ray beam enters the instrument from the right and after the sample the beam propagates through the flight tube which can be seen on the left. One small rack is required for the operation of LamNI and all electrical connections are interfaced on a quick-connect box at the setup, which allows the setup to be fully connected within minutes.

## 6. Positioning accuracy and stability   

**Figure 6:**
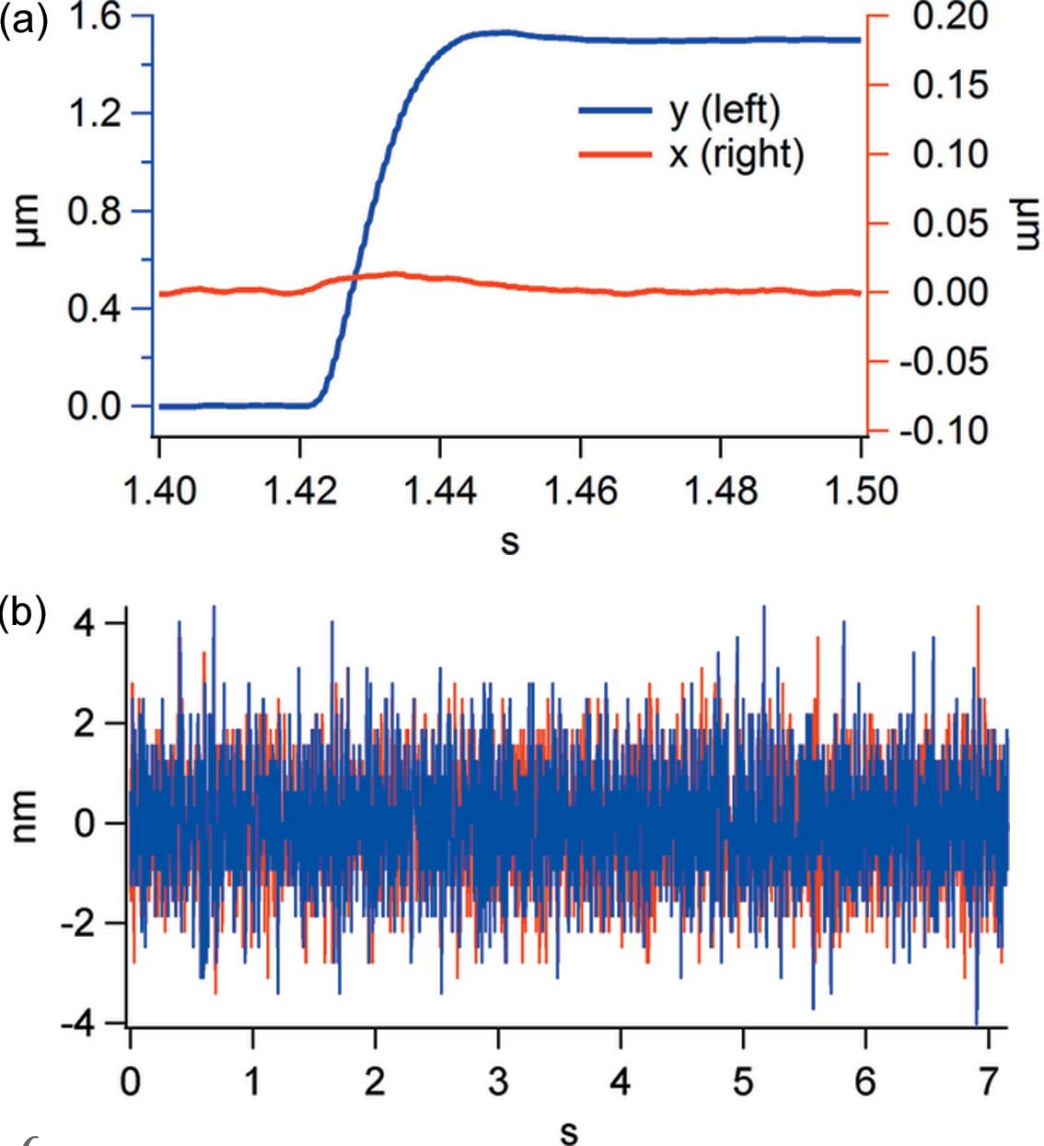
(*a*) Step response of LamNI, 40 ms for a 1.5 µm step. (*b*) In-position stability of LamNI with a 1.1 nm standard deviation.

For any scanning microscope, scanning overhead is an important issue. Concepts of continuous scanning trajectories exist (Odstrčil *et al.*, 2018*a* ▸; Pelz *et al.*, 2014 ▸; Deng *et al.*, 2015 ▸) that can help to overcome positioning overhead, but in these cases the imaging quality is reduced for a given dose, unless the continuous step is smaller than one resolution element (Odstrčil *et al.*, 2018*a* ▸), which comes with a steep increase in detector speed and computational requirements. To obtain high resolution and imaging quality in this instrument we use a step-scan; for this case high throughput is preserved by placing the sample downstream of the focus, which allows the use of large scanning steps. This can be further improved in the future by implementing a hybrid scanning mode (Odstrčil *et al.*, 2019*c* ▸). Fig. 6 ▸(*a*) depicts the interferometrically measured step response below 40 ms for a step size of 1.5 µm, which is typical in our imaging configuration. The positioning system was operated in closed loop mode using the interferometer positions for feedback, and the rotation stage was at 45°, such that for the depicted vertical step both axes of the Piezo scanner had to travel equal distances. The crosstalk with the *x* axis remains marginal.

Aside from the step response, an important factor is the in-position stability during exposure. While heavy structures result in low resonance frequencies, the amplitude of unwanted oscillations can become quite large. Special care has been taken in the design using a finite-element simulation of the base plate of LamNI, but also of the holders of the large sample stage to achieve a resonance frequency above 250 Hz. Fig. 6 ▸(*b*) shows the resulting in-position stability interferometrically measured between the sample and optics stage. The standard deviation is 1.1 nm in either direction.

**Figure 7:**
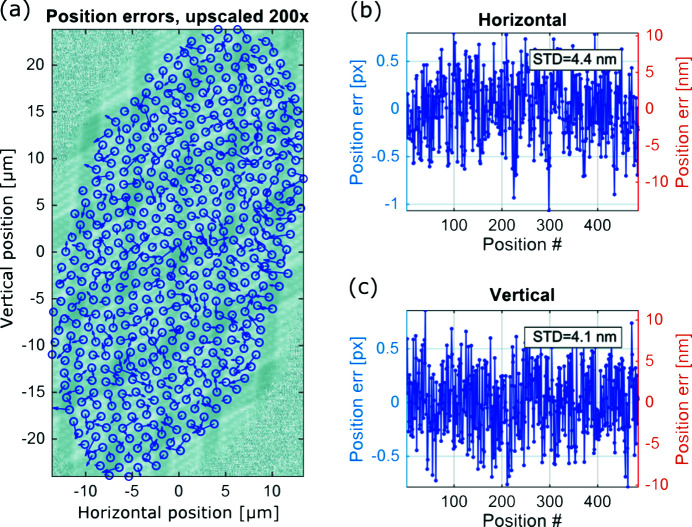
The accuracy of the interferometric system was verified by performing a ptychography scan of an integrated circuit sample and then applying a ptychographic position refinement algorithm. The standard deviation of the position errors in (*b*) and (*c*) was 4 nm for a reconstruction with a 13 nm pixel size. The position errors indicated by arrows in (*a*) are upscaled 200× in order to make them visible.

Positioning accuracy is ultimately confirmed using ptychographic position refinement (Odstrčil *et al.*, 2018*b* ▸) in an X-ray measurement as shown in Fig. 7 ▸. A ptychography scan of an integrated circuit sample was performed covering an area of 25 µm × 50 µm in the interferometer plane by defining a circular field of view in the object plane. Then the position refinement algorithm was used to estimate the position errors for each individual scan point, which are shown in Figs. 7 ▸(*b*) and 7(*c*). The standard deviations of the position errors in the horizontal and vertical directions are 4.4 nm and 4.1 nm, respectively.

## 7. Summary and application examples   

We have developed and built an instrument for performing ptychographic X-ray lamino­graphy. The total imaging area of 12 mm × 12 mm is covered by a direct scanning mechanism over 100 µm × 100 µm extended via coarse stepper motors and stitching. Positioning accuracy is achieved through dedicated differential laser interferometry measuring the relative position between the beam-defining FZP and the sample. For this metrology to be compatible with the lamino­graphic configuration and large imaging area, the required mirrors are held in position by a secondary anti-rotation mechanism. The system allows 3D nano-imaging of flat extended objects, thus simplifying sample preparation drastically for many systems. In addition, the region of interest can be freely chosen and need not be pre-determined at the sample preparation step as for computed nanotomography. As an application, the 3D measurement of an integrated circuit manufactured in 16 nm technology is presented by Holler *et al.* (2019 ▸), also demonstrating the zooming capabilities of the setup. The setup and measurement geometry have also proven suitable for 3D measurements with magnetic contrast. A first demonstration can be found in the work by Donnelly *et al.* (2020 ▸), which shows time-resolved magnetic lamino­graphy, a pump–probe technique, giving access to the temporal evolution of the magnetization in a 3D microdisc with 50 nm resolution, and with a synchrotron-limited temporal resolution of 70 ps.

## Supplementary Material

Video 1: Three synchronized views of the rotation stage rotating from 0 to 90 degrees. DOI: 10.1107/S1600577520003586/pp5154sup1.avi

Figure S1. DOI: 10.1107/S1600577520003586/pp5154sup2.pdf
