# Supplementary material for: LamNI – an instrument for X-ray scanning microscopy in lamino­graphy geometry
Source: J Synchrotron Radiat. 2020 Apr 6;27(Pt 3):730–6. doi: 10.1107/S1600577520003586 (PMC7206541; doi:10.1107/S1600577520003586)
Supplement: Supplementary file 2 [file s-27-00730-sup2.pdf]

JOURNAL OF SYNCHROTRON RADIATION

Volume 27 (2020)

Supporting information for article:

LamNI – an instrument for X-ray scanning microscopy in laminography geometry

Mirko Holler, Michal Odstrčil, Manuel Guizar-Sicairos, Maxime Lebugle, Ulrich Frommherz, Thierry Lachat, Oliver Bunk, Joerg Raabe and Gabriel Aeppli

LamNI – an instrument for X-ray scanning microscopy in laminography geometry

Supporting Figures

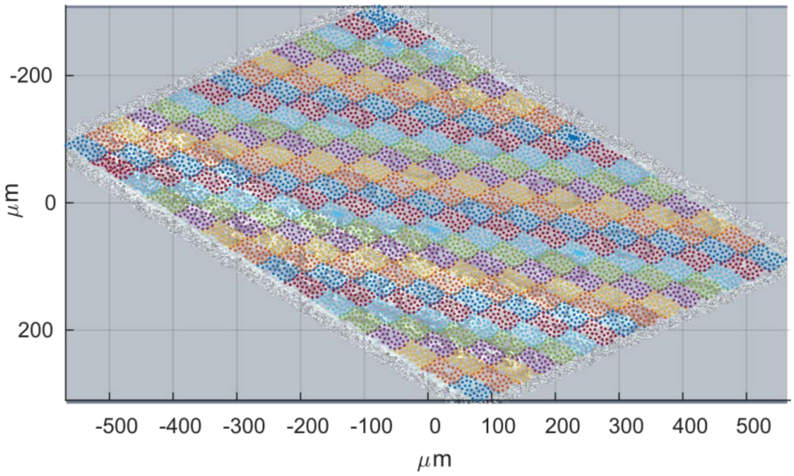

Figure S1 A demonstration of the stitching capability of the instrument. The figure shows a single projection with a square shape about $(0.5\text{mm})^2$ in size in the sample plane. The data was acquired by scanning many smaller areas using the Piezo stage and stitching such scans using the coarse stages. The individual sub-scans are indicated by differently colored symbols.
